# How much should you worry about contaminant neutrons in spatially fractionated grid radiation therapy?

**DOI:** 10.1371/journal.pone.0280433

**Published:** 2023-01-13

**Authors:** Farshid Mahmoudi, Najmeh Mohammadi, Meysam Haghighi, Zahra Alirezaei, Iraj Jabbari, Nahid Chegeni, Soheil Elmtalab, Hector Rene Vega-Carrillo, Ali Kazemian, Ghazale Geraily, Amir Hossein Karimi

**Affiliations:** 1 Razi Herbal Medicines Research Center, School of Allied Medical Sciences, Lorestan University of Medical Sciences, Khorramabad, Iran; 2 Department of Physics, Faculty of Science, Sahand University of Technology, Tabriz, Iran; 3 Neuroimaging and Analysis Group, Tehran University of Medical Sciences, Tehran, Iran; 4 School of Paramedicine, Bushehr University of Medical Sciences, Bushehr, Iran; 5 Department of Nuclear Engineering, Faculty of Physics, University of Isfahan, Isfahan, Iran; 6 Department of Medical Physics, School of Medicine, Ahvaz Jundishapur University of Medical Sciences, Ahvaz, Iran; 7 Department of Medical Physics and Biomedical Engineering, School of Medicine, Tehran University of Medical Sciences, Tehran, Iran; 8 Academic Unit of Nuclear Studies, University Autonomous of Zacatecas, Zacatecas, Mexico; 9 Radiation Oncology Research Center, Cancer Institute, Tehran University of Medical Sciences, Tehran, Iran; Chung-Ang University Gwangmyeong Hospital, REPUBLIC OF KOREA

## Abstract

Neutron contamination in radiation therapy is of concern in treatment with high-energy photons (> 10 MV). With the development of new radiotherapy modalities such as spatially fractionated grid radiation therapy (SFGRT) or briefly grid radiotherapy, more studies are required to evaluate the risks associated with neutron contamination. In 15 MV SFGRT, high-Z materials such as lead and cerrobend are used as the block on the tray of linear accelerator (linac) which can probably increase the photoneutron production. On the other hand, the high-dose fractions (10–20 Gy) used in SFGRT can induce high neutron contamination. The current study was devoted to addressing these concerns via compression of neutron fluence (Φ_n_) and ambient dose equivalent ($H_{n}^{*}(10)$) at the patient table and inside the maze between SFGRT and conventional fractionated radiation therapy (CFRT). The main components of the 15 MV Siemens Primus equipped with different grids and located inside a typical radiotherapy bunker were simulated by the MCNPX^®^ Monte Carlo code. Evidence showed that the material used for grid construction does not significantly increase neutron contamination inside the maze. However, at the end of the maze, neutron contamination in SFGRT is significantly higher than in CFRT. In this regard, a delay time of 15 minutes after SFGRT is recommended for all radiotherapy staff before entering the maze. It can be also concluded that $H_{n}^{*}(10)$ at the patient table is at least 10 times more pronounced than inside the maze. Therefore, the patient is more at risk of neutrons compared to the staff. The $H_{n}^{*}(10)$ at the isocenter in SFGRT with grids made of lead and cerrobend was nearly equal to CFRT. Nevertheless, it was dramatically lower than in CFRT by 30% if the brass grid is used. Accordingly, SFGRT with the brass grid is recommended, from radiation protection aspects.

## 1. Introduction

Nowadays, radiotherapy of deeply-seated tumors using high-energy photons is known as an efficient technique for the treatment of cancer patients. High-energy photons are generated through medical linac in most of the radiation therapy clinics. Neutron contamination is of concern in the safe usage of high-energy photons [1–3]. The linac head is mainly composed of high atomic number elements such as lead, tungsten (shielding and collimators), and gold (target), which leads to a significant portion of photoneutron production, through (γ, n) reaction [4]. Based on the evidence, an 18 MV Varian 2100 C/D can produce 1.38 × 10^12^ neutrons per 1 Gy photon dose delivered to the isocenter (IC) [5]. Since the threshold energy for (γ, n) reaction is at least 8 MeV [6], neutron contamination in radiation therapy is of concerns in treatment with high-energy photons (> 10 MV). Recently, many researchers in the field of radiation protection have addressed the role and consequences of unwanted doses to the patient caused by photoneutrons [7–9]. Some efforts focused on reducing neutron production in the linac head through optimization of the Bremsstrahlung target [10]. Considering that the neutron is an uncharged particle that is difficult to detect, it is important to determine the neutron spectrum (Φ_n_(E)) and the ambient dose equivalent ($H_{n}^{*}(10)$) to estimate the effective dose received by radiotherapy patients and staff.

Evidence shows that the average energy of the photoneutrons at the patient table is approximately 1 MeV [9]. The radiation weighting factor for such neutrons was estimated to be about 20 based on the international commission on radiological protection (ICRP) formula in report 103 [11]. It means that such photoneutrons can deliver a dose nearly 20 times larger than diagnostic photons. The contribution of contaminant neutrons in out-of-field dose is sometimes more pronounced than scattered photons. For example, the unwanted dose to the breasts of a patient undergoing 18 MV pelvic radiotherapy originates from photoneutrons by 67% [12]. Additionally, it has been shown that for glioma and hepatocellular patients undergoing 18 MV radiotherapy, secondary fatal cancer risk due to neutron contamination can reach 281 and 844 persons per one million persons, respectively [7, 9]. For prostate patients undergoing 18 MV radiotherapy, it was reported that the total risk of secondary cancer in eye lenses, thyroid, and chiasma is 870 persons per one million persons [8]. Considering the risks of neutron contamination in radiotherapy, further studies are required to evaluate the level of neutron contamination in new radiotherapy modalities (such as SFGRT).

SFGRT has been recently suggested as a new modality for the treatment of large tumors (>8 cm in diameter) not responding well to conventional doses (2 Gy/session) [13]. The most important achievement at the therapeutic level is the ability to deliver a single high-dose fraction (10–20 Gy) without significant complications to the normal tissue surrounding the tumor [14, 15]. In SFGRT, the therapeutic beam is divided into several small circular fields by installing a block called the grid on the linac tray. Contrary to the common belief, cell death still occurs in the blocked regions. The main mechanism of cell death in the blocked regions is considered to be the bystander effect [16]. Clinical achievements of high-energy SFGRT have been discussed in detail in the literature [17–19].

In SFGRT, high-Z materials such as lead and cerrobend are used as the grid on the linac tray which have the potential to increase the photoneutron production [9]. On the other hand, the high-dose fractions (10–20 Gy) used in SFGRT can induce high neutron contamination. Nevertheless, Wang et al. [20] demonstrated that for a constant monitor unit (MU), neutron equivalent dose to the patient in 18 MV SFGRT is, on average, 35% less noticeable than CFRT. From radiation protection aspects it was recommended that comparisons of neutron contamination between SFGRT and CFRT should be performed based on 1 Gy photon dose delivered to the depth of maximum dose (d_max_) [9]. Accordingly, Karimi et al. [9] showed that the Φ_n_ inside the large field sizes for SFGRT with lead block is, on average, 23% higher than in CFRT. A similar analysis on neutron contamination inside the maze of radiotherapy room showed that neutron ambient dose equivalent, $H_{n}^{*}(10)$, was up to 50% higher than in CFRT [21].

From the perspective of radiation protection, SFGRT with 15 MV photons was proposed recently as an alternative [9]. However, it is still doubtful since there is no information in the literature to reflect the severity of neutron contamination in 15 MV SFGRT compared to CFRT. Accordingly, the authors were encouraged to address this concern with the evaluation of neutron spectrum, $H_{n}^{*}(10)$, and Φ_n_ inside a typical radiotherapy room. For this aim, Monte Carlo (MC) simulation was employed as a precise tool for neutron spectrometry.

## 2. Materials and methods

### 2.1 Ethics statement

The authors declare that this study does not involve human participants and only reports data obtained via in-vitro dosimetry. Therefore, participant consent was not requested in the research.

### 2.2 Monte Carlo simulation

The main challenge for neutron dosimetry in the mixed fields, i.e. the fields containing a significant proportion of photon flux, is the detector saturation [22]. Due to the inherent sensitivity of the most neutron detectors to photons, the separation and detection of neutrons is a tough process, especially when the neutron flux is low. It often leads to at least 10% uncertainty in measurements [23]. In addition, measurement of the neutron dose without knowledge of the neutron spectrum leads to up to 20% measurement uncertainty [24]. Thus, MC simulation was recommended as an accurate and efficient tool to estimate neutron contamination in radiation therapy [24]. To this end, simulation geometry and cross-section libraries for physical interactions need a strong benchmark.

In this study, the main components of the 15 MV Siemens Primus head, including the target, absorbers, flattening filters, primary collimator, ionizing chamber, jaws, bending magnet, and shield, were simulated by the MCNPX^®^ code (2.7.0 extensions) [25]. This model had been previously benchmarked by Mohammadi et al. [26] through comparing the percent depth doses (PDDs) and dose profiles obtained from the measurements with those extracted from MC calculations. They found that the geometry and the cross-section libraries complied well with the requirements. By optimizing the energy distribution of electrons (13.76 MeV) impinging on the Bremsstrahlung target of the linac, the difference is below 2% between the measured and the MC results. In this study, the EL03, MCPLIB04, and ENDFB-VII cross-section libraries were used to simulate electron, photons, and neutron interactions with matter, respectively. Photoneutron production was simulated by LA150u, KAREI01u, and CNDC01u cross-section libraries [25].

**Fig 1:**
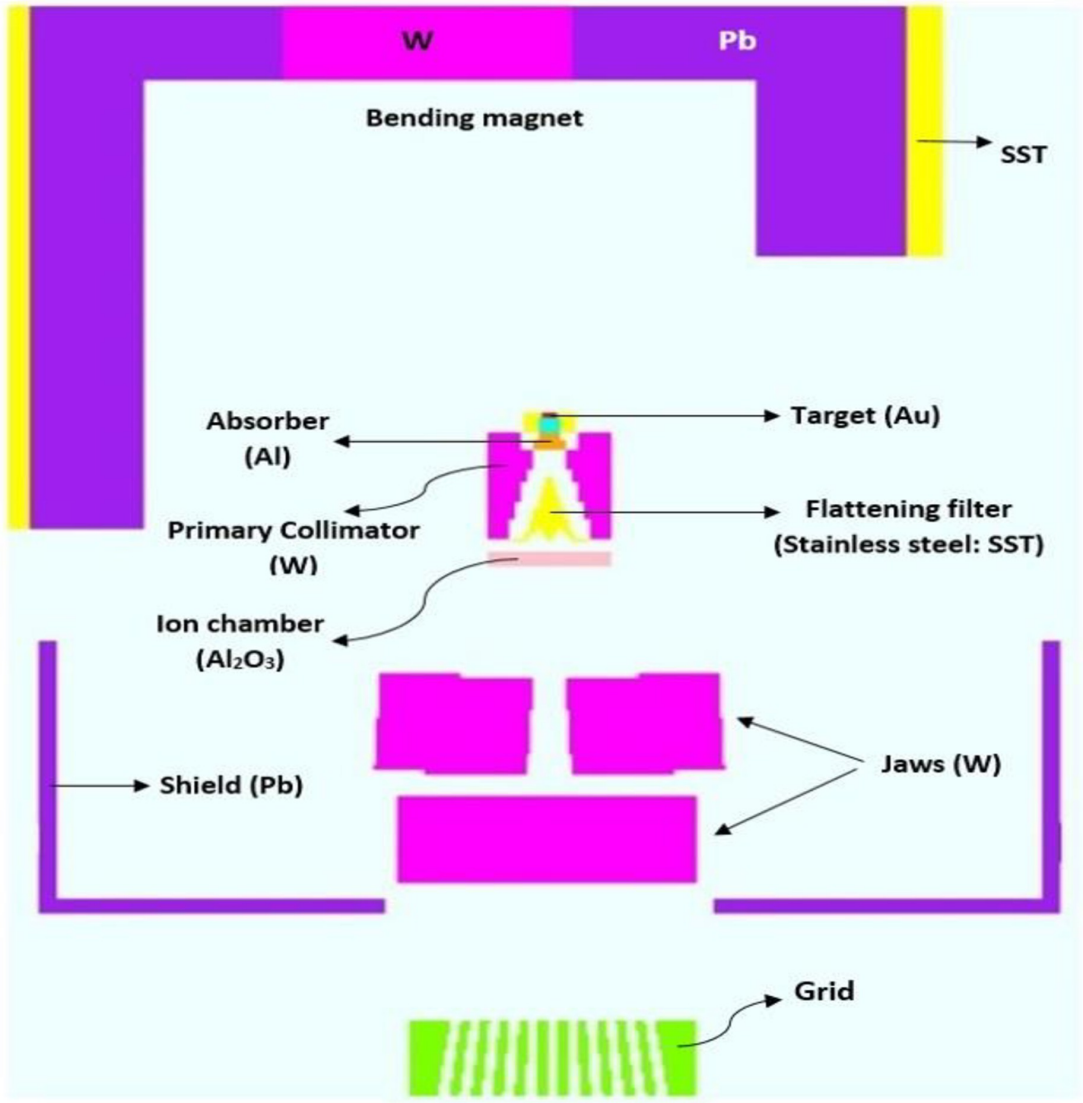
Siemens Primus in 15 MV mode simulated by MCNPX^®^.

The grids were modeled based on the commercial pattern which belongs to dot Decimal^®^ company (Sanford, FL 32771, USA). The grid consists of 127 holes arranged hexagonally and divergent the photon beam. The holes project circular fields with a diameter of 1 cm and a center-to-center distance of 2 cm at 100 cm far away from the Bremsstrahlung target. Using this pattern, a maximum field size of 25 × 25 cm^2^ at IC can be irradiated with the grid. Nevertheless, all calculations in this study were performed under the 10 × 10 cm^2^ field size as a common treatment field in both CFRT and SFGRT. Cerrobend, lead and brass were used to model the grids. Fig 1 presents the geometry simulated by MCNPX^®^.

**Fig 2:**
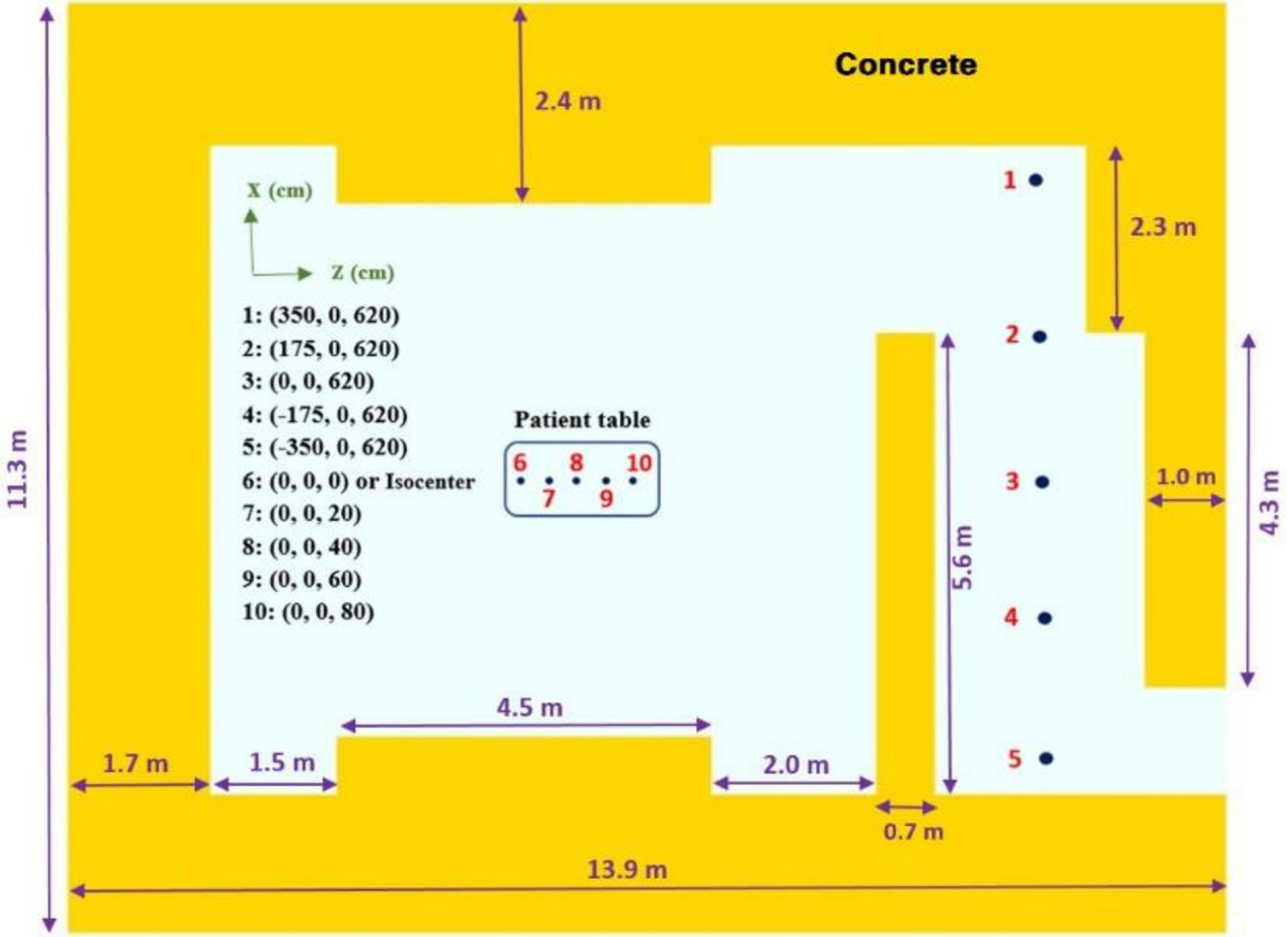
Scheme of a typical radiotherapy room simulated by MCNPX^®^. The thicknesses of the floor and ceiling were 1.0 m and 1.7 m, respectively.

To compare the spectrum of neutron contamination between two photon beams (15 MV vs. 18 MV) under a similar condition, the bunker used by Karimi and Vega-Carrillo [21] was modeled as is shown in Fig 2. The density of 2.35 g/cm^3^ was considered for the concrete walls composed by O (49.83%), Si (31.58%), Ca (8.26%), Al (4.56%), K (1.92%), Na (1.71%), Fe (1.22%), and H (0.92%).

International commission on radiation units and measurements (ICRU) defines the $H_{n}^{*}(10)$ as the neutron dose equivalent delivered by the corresponding expanded and aligned field in the ICRU sphere to the depth of 10 mm, on the radius opposing the direction of the field [27]. To evaluate the $H_{n}^{*}(10)$ and the neutron spectra, inside the maze and at the patient table, 5 and 10 cm radius point-detectors (tally f5) were employed, respectively. Finally, using the neutron fluence-to-dose conversion coefficients from report 74 of the ICRP [28], the $H_{n}^{*}(10)$ was calculated for the indicated locations enumerated in Fig 2. The detectors have the potential to calculate neutron spectra (Φ_n_(E)), as well. Accordingly, Φ_n_(E) at the IC and at the end of the maze (detector 5) were estimated from 10^−9^ to 10 MeV in 100 logarithmic bins. It is worthwhile to be mentioned that the MCNPX^®^ results are normalized to the initial particle (in this case is per electron or per history). Therefore, to report the results based on the 1 Gy photon dose delivered to d_max_, the photon dose absorbed at d_max_ was also calculated in terms of Gy/electron history using mesh tally type 1 (energy deposition). To compare photon dose distribution between SFGRT and CFRT, the two-dimensional dose profile in both open and grid fields was calculated using mesh tally type 1 inside the water phantom at a depth of 5 cm. To keep the relative errors of MC calculations within 3% (in most cases), up to 3 × 10^9^ electron histories were traced in the simulations. For more information about the relative errors, the readers are referred to the S1–S5 Files.

## 3. Results

**Fig 3:**
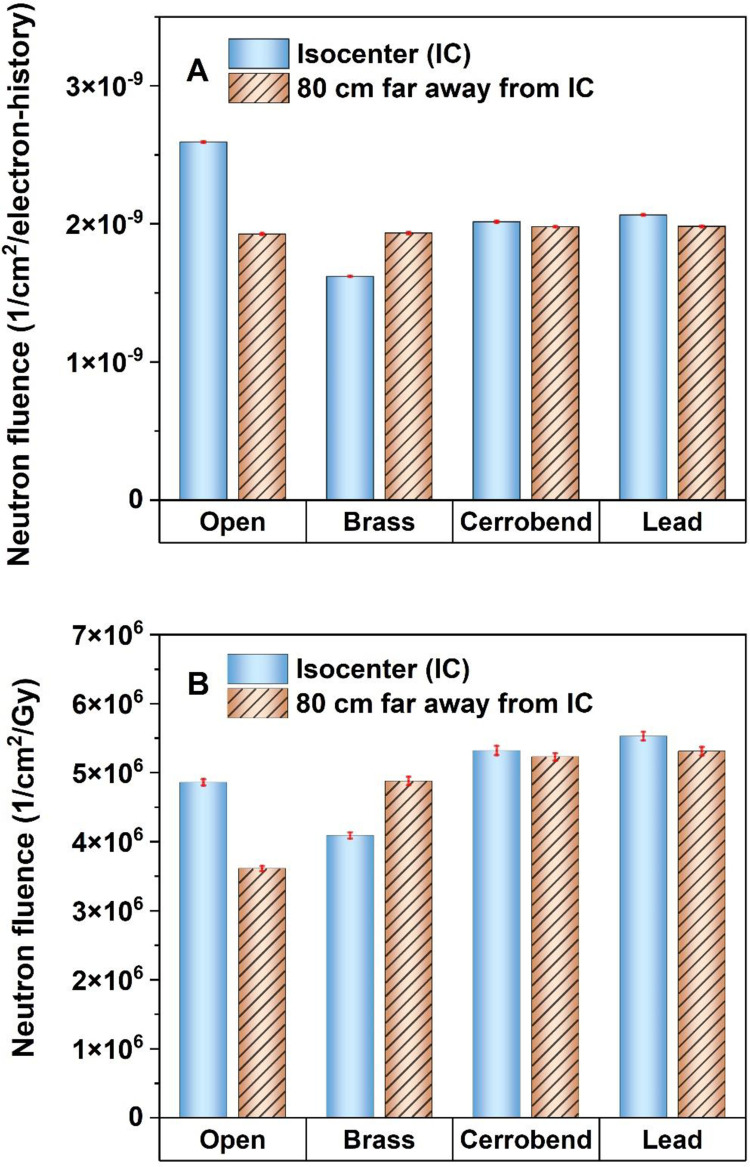
Comparison of neutron fluence at the patient table between conventional radiotherapy (open field) and grid radiotherapy (using different grids made of brass, cerrobend, and lead). The comparison was based on 1 electron history impinging on the Bremsstrahlung target (A) and 1 Gy photon dose delivered to d_max_ (B).

The comparison of the Φ_n_ at the IC and at 80 cm far away from the IC between open and grid fields was illustrated in Fig 3. These comparisons were made based on two scenarios as follows: scenario A in which data is reported based on 1 electron history impinging on the Bremsstrahlung target. This procedure leads to a conclusion similar to the circumstances in which a constant MU is used for the dose delivery both for open and grid fields; scenario B in which the data is reported based on 1 Gy photon dose delivered to d_max_.

**Fig 4:**
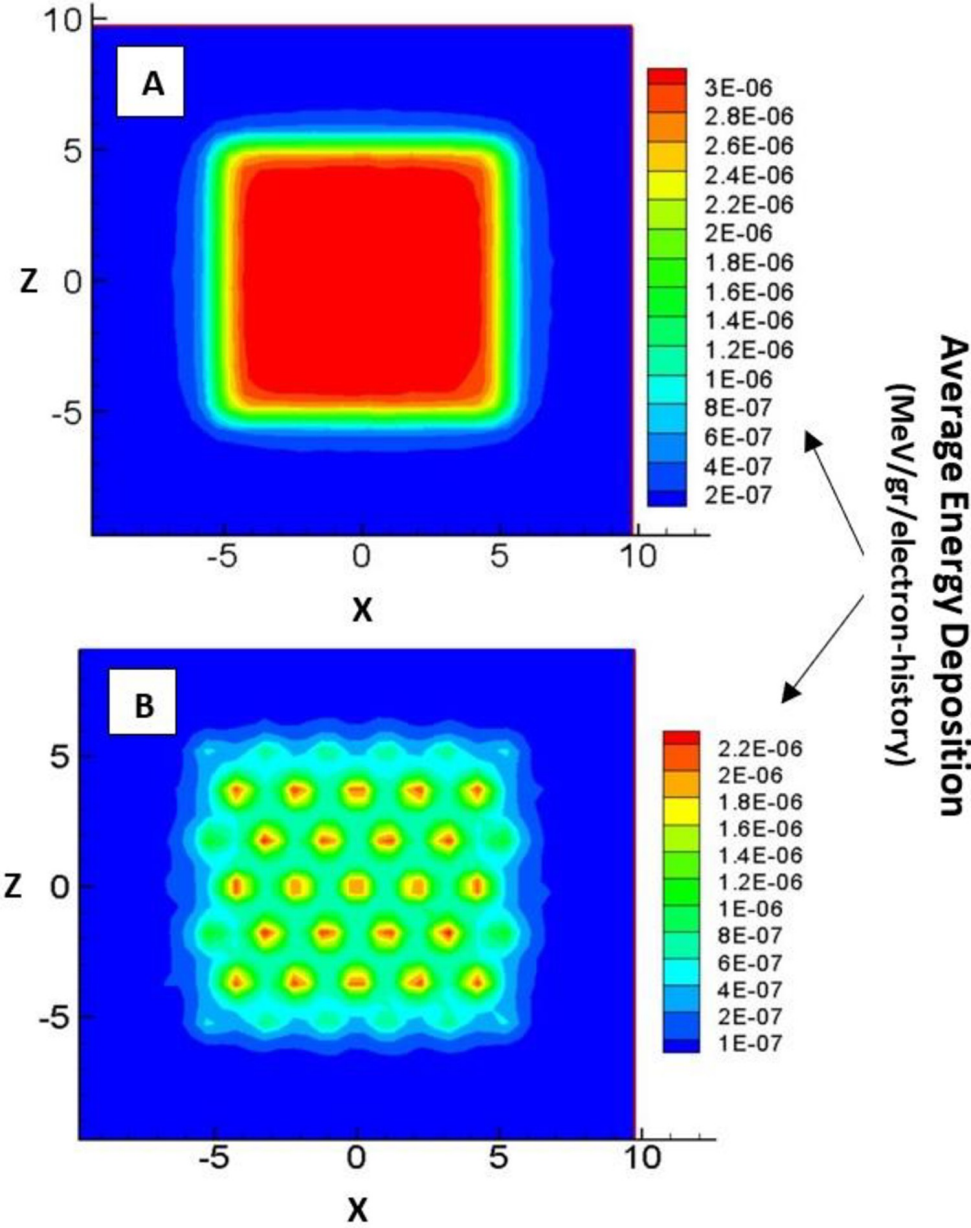
Two-dimensional dose profile at a depth of 5 cm inside the water phantom for the open field (A) and the field blocked by a brass grid (B).

The photon dose absorbed at d_max_ in the open field was 5.34 × 10^−16^ Gy/electron history. In the case of fields blocked by grids made of brass, cerrobend, and lead, it decreased to 3.96 × 10^−16^, 3.79 × 10^−16^, and 3.73 × 10^−16^ (all in terms of Gy/electron history), respectively. Fig 4 shows the two-dimensional profile of the dose at a depth of 5 cm for the open field and the field blocked by the brass grid as well.

**Fig 5:**
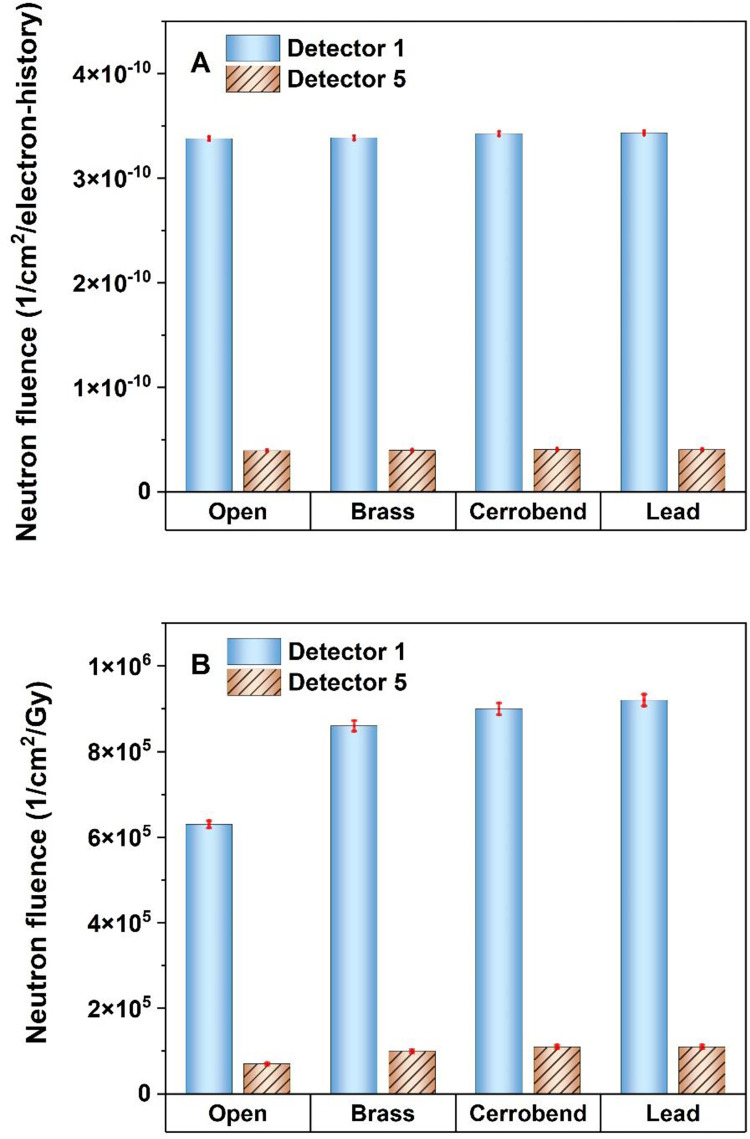
Comparison of neutron fluence inside the maze between conventional radiotherapy (open field) and grid radiotherapy (using different grids made of brass, cerrobend, and lead). The comparison was based on 1 electron history impinging on the Bremsstrahlung target (A) and 1 Gy photon dose delivered to d_max_ (B).

In Fig 5, neutron fluence under open and grid fields was analyzed inside the maze based on 1 electron-history impinging on the Bremsstrahlung target and 1 Gy photon dose delivered to d_max_ as well.

**Fig 6:**
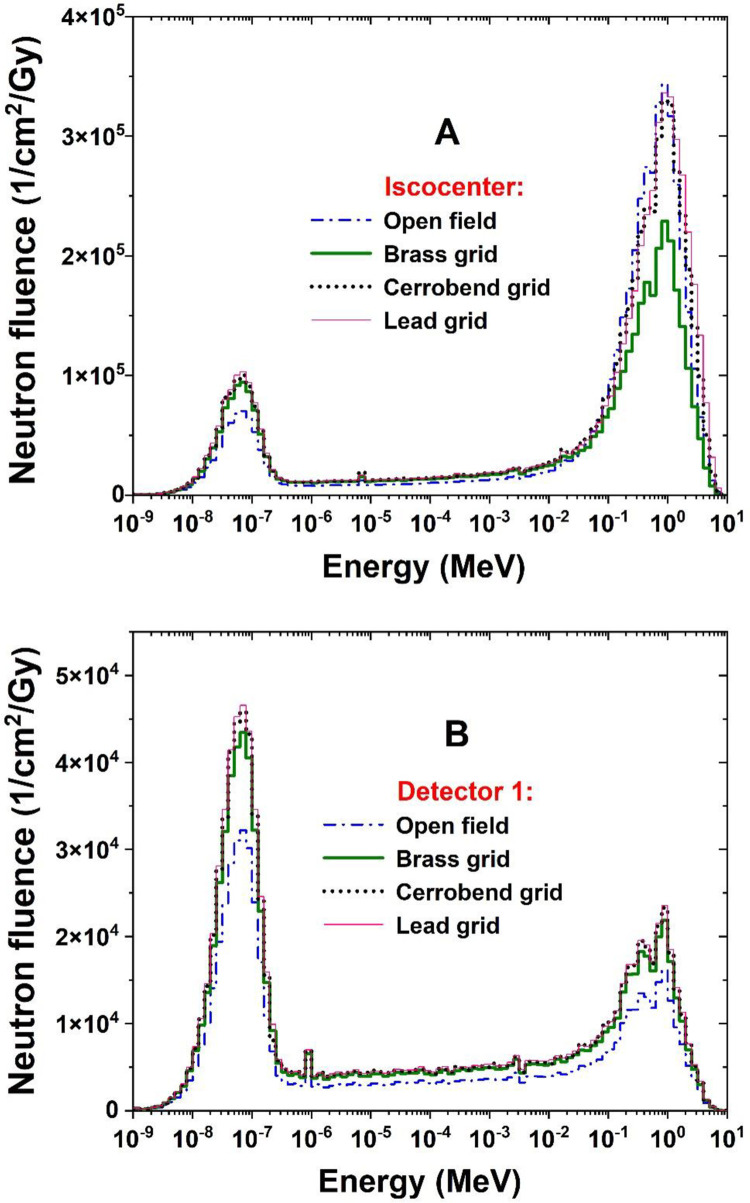
Comparison of neutron spectra at the isocenter (A) and at the end of the maze (B) between conventional radiotherapy (open field) and grid radiotherapy (using different grids made of brass, cerrobend, and lead).

In Fig 6, the comparison of the neutron energy spectra at the IC and also at the end of the maze (detector 1) was shown for both the open and the grid fields.

Table 1 reports the Φ_n_ and $H_{n}^{*}(10)$ inside the maze for the open and grid fields under 15 MV radiotherapy.

**Table 1:** Neutron fluence (*Φ*_*n*_) and neutron ambient dose equivalent ($H_{n}^{*}$ (10)) inside the maze in 15 MV radiotherapy.

Detector	Φ_n_ [10^5^/cm^2^/Gy]					$H_{n}^{*}(10)$ [μSv/Gy]		
	Conventional radiotherapy	Grid radiotherapy			Conventional radiotherapy	Grid radiotherapy		
		Brass	Cerrobend	Lead		Brass	Cerrobend	Lead
**1**	6.3	8.6	9.0	9.2	51.1	69.2	73.3	74.8
**2**	3.8	5.2	5.5	5.6	17.9	24.2	25.7	26.2
**3**	1.7	2.3	2.4	2.4	5.7	7.7	8.2	8.4
**4**	1.0	1.3	1.4	1.4	2.9	3.9	4.1	4.2
**5**	0.7	1.0	1.1	1.1	1.9	2.6	2.7	2.7

Table 2 quantifies the $H_{n}^{*}(10)$ at the patient table under open field and different grid fields when the 15 MV photon beam is used for dose delivery.

**Table 2:** Neutron ambient dose equivalent, $H_{n}^{*}$ (10), at the patient table in 15 MV radiotherapy.

Detector	$H_{n}^{*}(10)$ [mSv/Gy]			
	Conventional radiotherapy	Grid radiotherapy		
		Brass	Cerrobend	Lead
**6**	1.06	0.71	1.09	1.18
**7**	0.79	0.91	1.15	1.21
**8**	0.67	0.91	1.04	1.08
**9**	0.73	1.00	1.08	1.11
**10**	0.71	0.97	1.04	1.06

## 4. Discussion

Two scenarios can potentially increase neutron contamination in SFGRT compared to CFRT. First, interaction of the grid with high-energy photons as a new source for photoneutron production. Second, more MUs which are probably used to deliver 1 Gy of photon dose to d_max_.

### 4.1 Grid as a barrier for contaminant neutrons

Fig 3A reveals that for a constant MU, the Φ_n_ at the IC in 15 MV SFGRT with brass, cerrobend, and lead grids, is respectively 38%, 22%, and 20% lower than in the open field (CFRT), which is comparable with 55%, 31%, and 31% reported for 18 MV SFGRT [9]. In this regard, Chegeni et al. reported that in 18 MV SFGRT, a brass grid can reduce Φ_n_ at the IC by a factor of 48% in comparison with CFRT [29]. Fig 3A confirms that the grid acts as a barrier and, as a result, weakens the photoneutrons produced by the upper parts of the linac head. This effect is only visible at the IC since it is directly under the grid. So, at a distance of 80 cm far away from the IC, there was no significant difference in the Φ_n_ between open and grid fields. Accordingly, the first hypothesis is rejected in which the grid cannot be considered a new source of photoneutron production despite the high-Z materials used for its construction. This finding confirms the study conducted by Karimi et al. on 18 MV SFGRT [9]. Although this approach is helpful in specifying the role of the grid as a barrier or a new source for photoneutron production in the linac head, it is not beneficial from the radiation protection point of view.

### 4.2 Monitor units required for delivery of 1 Gy photon dose to d_max_

The photon dose absorbed at d_max_ in the open field was 5.34 × 10^−16^ Gy/electron history while for the blocked fields by brass, cerrobend, and lead grids, it decreased to 3.96 × 10^−16^, 3.79 × 10^−16^, and 3.73 × 10^−16^ (all in terms of Gy/electron history), respectively. It means that in comparison with open field, more MUs (roughly 1.5x) are required to deliver 1 Gy photon dose to d_max_ because the grids block the photon beam with a fraction of 50%. This finding introduces Scenario 2 as the main reason for more neutron contamination in SFGRT compared to CFRT. This finding also can be confirmed via a 2-D graphical view of the dose distribution. In the regions blocked by a brass grid (Fig 4B), the dose distribution is almost uniform. Nevertheless, it is still lower than in the open field (Fig 4A). As to the circular small fields arranged hexagonally, the dose decreases gradually with increasing distance from the center. The reason is scattering in the edge of the field which leads to electron imbalance. If the resolution of mesh tallies (5 × 5 × 1 mm^3^) in dose calculation were smaller than the current value, dose modulation in the grid fields could be observed more clearly compared to the open field.

### 4.3 Intensifying contaminant neutrons in the grid fields

As mentioned in the previous section, in SFGRT more MUs are required to deliver 1 Gy of photon dose to d_max_ which may result in producing more contaminant photoneutrons compared to CFRT (Scenario 2). Therefore, it is more meaningful to make comparisons in terms of delivery of 1 Gy photon dose to d_max_ in each modality (Fig 3B). Based on the evidence illustrated in Fig 3B, the Φ_n_ at the IC in SFGRT with the brass grid was estimated to be 15% lower than in CFRT. Additionally, it was approximately 10% higher than CFRT when cerrobend or lead grids were used as the block in the simulations. A similar analysis was done at a distance of 80 cm far away from the IC, which showed that the Φ_n_ with the grid is, on average, 40% higher than in CFRT. Consequently, evaluation of the out-of-field dose caused by photoneutrons in 15 MV SFGRT should be of interest in future studies.

As it was shown in Fig 3B, the Φ_n_ at the IC for the fields blocked by cerrobend or lead grids is almost 30% more than in the brass grid. The reason is both a higher cross-section of Pb compared to Cu and Sn for (γ, n) interaction [30] and more MUs needed for delivering 1 Gy photon dose to d_max_.

### 4.4 Neutron spectrometry inside the maze

Evaluation of neutron contamination inside the maze is important to protect radiotherapy staff entering the maze. Fig 5A showed that Φ_n_ inside the maze is independent of both treatment modality (SFGRT vs. CFRT) and the type of grid. In other words, for a constant MU or electron history impinging on the Bremsstrahlung target, the Φ_n_ takes approximately the same value for both SFGRT and CFRT. Though, by converting the data based on 1 Gy photon dose delivered to d_max_ in each modality (Fig 5B), the Φ_n_ at the end of the maze (detector 1) in SFGRT was almost 40% more than in CFRT. The situation is different at the entrance of the maze (detector 5), and no significant difference in the Φ_n_ was found between SFGRT and CFRT. This is due to the successive attenuation of the photoneutrons along the maze.

Fig 6A shows the neutron energy spectrum at the IC for the open and the grid fields. The appearance of the spectrum is similar to the corresponding cases in Karimi and Vega-Carrillo’s study on 18 MV photons [21]. It is noticeable that there are two peaks, one related to the fast neutrons (the higher peak) and the other related to the thermal neutrons (the shorter peak). The average energy extracted from these spectra was estimated to be 537 keV for the open field and 430, 553, and 619 keV for the grid fields blocked by brass, cerrobend, and lead grids, respectively. If these values are applied in the ICRP exponential formula [11], one can find the constant value of 19 ± 1 for W_R_ of contaminant neutrons. It means there is no difference in the destructive power of photoneutrons produced in SFGRT and CFRT. Given that more neutrons are produced in the grid fields (Fig 3A), evaluation of neutron equivalent dose, secondary cancer risk, and risk of genetic effects could be of interest for future studies.

Fig 6B shows that compared to the IC, the neutron spectrum at the end of the maze (detector 1) was greatly modulated. The height of the thermal peak has grown significantly, especially in SFGRT; on the other hand, the fast peak tends to decrease. The average neutron energy under these conditions was 166 ± 1 keV for both open and grid fields. It can be concluded that neutron average energy inside the maze is independent of the treatment modality (SFGRT vs. CFRT). As a result, the authors suggest that in order to monitor neutron contamination inside the maze, neutron detectors should be calibrated under the same conditions.

### 4.5 Ambient dose equivalent of contaminant neutrons

From Table 1, it can be observed that Φ_n_ and $H_{n}^{*}(10)$ along the maze reduce nearly by a factor of 0.5 for both SFGRT and CFRT which is comparable well with the factor of 0.6 in Waller et al.’s study on 18 MV photons [31]. This finding implies that the extreme attenuation of neutrons in interaction with the concrete walls is the main reason for approximately unchanged value of Φ_n_ at detector 5 (Fig 5B). It is worthwhile to mention that Φ_n_ and $H_{n}^{*}(10)$ in SFGRT with the lead or cerrobend grid is only, on average, 7% higher than in SFGRT with the brass grid. This difference is ignorable regarding the relative error of MC calculations (3%). Therefore, the material used for grid construction does not significantly increase neutron contamination inside the maze. Nevertheless, independent of detector location, Φ_n_ and $H_{n}^{*}(10)$ at the end of the maze (detector 1) in SFGRT is nearly 1.5 times that of CFRT. In comparison with data reported by Karimi and Vega-Carrillo’s study on 18 MV photons [21], it can be found that independent of treatment modality and detector location, Φ_n_ and $H_{n}^{*}(10)$ in SFGRT with 15 MV photons is almost 70% lower than in 18 MV photons. Therefore, given the concerns about 18 MV SFGRT, 15 MV SFGRT can be considered as an alternative from the radiation protection point of view. However, as Table 1 shows, neutron contamination at the end of the maze (detector 1) is nearly 1.5 times that of CFRT. Given that in SFGRT, the dose fraction is up to 10 times that of CFRT (20 Gy vs. 2 Gy), it is expected that for a single session of SFGRT, neutron contamination inside the maze will be 15 times more pronounced than in CFRT. Considering that ^28^Al with a half-life of 2.3 minutes is one the most critical radionuclides produced through ^27^Al(n, γ)^28^Al in routine CFRT [32], a delay time of 15 minutes after SFGRT is recommended for all radiotherapy staff before entering the maze. The authors suggest more studies in the future to benchmark this delay time.

Regarding to Tables 1 and 2, $H_{n}^{*}(10)$ at the patient table is at least 10 times more pronounced than inside the maze. Therefore, the patient is at risk of neutrons more than staff entering the maze. Table 2 explained that $H_{n}^{*}(10)$ at the IC in SFGRT with lead and cerrobend grids is nearly equal to CFRT. Nevertheless, it is dramatically 30% lower than in CFRT if the brass grid is used. This is due to the lower cross-section of (γ, n) reaction for the brass compared to lead [30], fewer MUs required to deliver 1 Gy photon dose to d_max_, and finally the effective role of the brass in the attenuation of photoneutrons (Fig 3A). For out-of-field distances, $H_{n}^{*}(10)$ in SFGRT with brass, cerrobend, and lead grids is, on average, 30%, 49%, and 54%, respectively, more than in CFRT. Given that the grid has no effective role in the neutron contamination inside the maze, SFGRT with the brass grid is recommended instead of lead or cerrobend grid, from radiation protection aspects.

## 5. Conclusion

We used Monte Carlo simulation to compare neutron spectra and $H_{n}^{*}(10)$ at the patient table and inside the maze in 15 MV radiotherapy between open and grid fields. Three types of grids made of brass, cerrobend, and lead were considered in the simulations. Evidence showed that the material used for grid construction does not significantly affect increasing neutron contamination inside the maze. In comparison with the literature, Φ_n_ and $H_{n}^{*}(10)$ in SFGRT with 15 MV photons is significantly lower than in 18 MV photons. Therefore, considering the common concerns, SFGRT 15 MV can be considered as an alternative from a radiation safety perspective. However, at the end of the maze, neutron contamination in SFGRT is significantly higher than in CFRT. In this regard, a delay time of 15 minutes after SFGRT is recommended for all radiotherapy staff before entering the maze.

We can also conclude that $H_{n}^{*}(10)$ at the patient table is at least 10 times that of the maze. Therefore, the risk of neutrons for the patient is more pronounced than for the employees entering the maze. Additionally, $H_{n}^{*}(10)$ at the IC in SFGRT with lead and cerrobend grids is nearly equal to CFRT. However, when the brass grid was used, $H_{n}^{*}(10)$ was dramatically lower than CFRT by 30%. Therefore, a brass grid is recommended for SFGRT, rather than a lead or cerrobend grid. For out-of-field distances, $H_{n}^{*}(10)$ in SFGRT was higher than in CFRT. Therefore, evaluation of the out-of-field dose caused by photoneutrons in 15 MV SFGRT may be of interest in future studies.

## Supporting information

S1 File Data set. (XLSX)

S2 File MCNPX^®^ output file for evaluation of neutron contamination in conventional radiotherapy. (O)

S3 File MCNPX^®^ output file for evaluation of neutron contamination in grid radiotherapy using a brass block. (O)

S4 File MCNPX^®^ output file for evaluation of neutron contamination in grid radiotherapy using a cerrobend block. (O)

S5 File MCNPX^®^ output file for evaluation of neutron contamination in grid radiotherapy using a lead block. (O)

S6 File Ethics code. (PDF)
