# Supplementary material for: How much should you worry about contaminant neutrons in spatially fractionated grid radiation therapy?
Source: PLoS One. 2023 Jan 13;18(1):e0280433. doi: 10.1371/journal.pone.0280433 (PMC9838865; doi:10.1371/journal.pone.0280433)
Supplement: S6 File — (PDF) [file pone.0280433.s006.pdf]

Tehran University of Medical Sciences

School of Medicine- Tehran University of Medical Sciences

Research Ethics Committees Certificate

Approval ID:	IR.TUMS.MEDICINE.REC.1401.644	Approval Date:	2023-01-04
Evaluated by:	Research Ethics Committees of School of Medicine- Tehran University of Medical Sciences		
Status:	Approved		
Approval Statement:	The project was found to be in accordance to the ethical principles and the national norms and standards for conducting Medical Research in Iran.<br>Notice:<br>1. Although the proposal has been approved by the Biomedical Research Ethics Committee, meeting the professional and legal requirements is the sole responsibility of the PI and other project collaborators.<br>2. This certificate is reliant on the proposal/documents received by this committee on 2023-01-04. The committee must be notified by the PI as soon as the proposal/documents are modified.		
Proposal Title:	Spectroscopy of contaminant neutrons in 15-MV Spatially Fractionated Grid Radiation Therapy (SFGRT)		
Principal Investigator:	Name: Ghazaleh Geraily<br>Email: ghazalegraily@yahoo.com		

Dr. Nima Rezaei
Committee Director
School of Medicine- Tehran University of Medical Sciences

Dr. Mahshad Khodarahmian
Committee Secretary
School of Medicine- Tehran University of Medical Sciences
